# Reduction of Genetic Diversity of the Harpy Eagle in Brazilian Tropical Forests

**DOI:** 10.1371/journal.pone.0148902

**Published:** 2016-02-12

**Authors:** Aureo Banhos, Tomas Hrbek, Tânia M. Sanaiotti, Izeni Pires Farias

**Affiliations:** 1 Departamento de Biologia, Centro de Ciências Exatas, Naturais e da Saúde, Universidade Federal do Espírito Santo, Alegre, Espírito Santo, Brazil; 2 Laboratório de Evolução e Genética Animal, Departamento de Genética, Instituto de Ciências Biológicas, Universidade Federal do Amazonas, Manaus, Amazonas, Brazil; 3 Coordenação de Biodiversidade, Instituto Nacional de Pesquisas da Amazônia, Manaus, Amazonas, Brazil; 4 Programa de Conservação do Gavião-real, Instituto Nacional de Pesquisas da Amazônia, Manaus, Amazonas, Brazil; Centre for Cellular and Molecular Biology, INDIA

## Abstract

Habitat loss and fragmentation intensify the effects of genetic drift and endogamy, reducing genetic variability of populations with serious consequences for wildlife conservation. The Harpy Eagle (*Harpia harpyja*) is a forest dwelling species that is considered near threatened and suffers from habitat loss in the forests of the Neotropical region. In this study, 72 historical and current samples were assessed using eight autosomal microsatellite markers to investigate the distribution of genetic diversity of the Harpy Eagle of the Amazonian and Atlantic forests in Brazil. The results showed that the genetic diversity of Harpy Eagle decreased in the regions where deforestation is intense in the southern Amazon and Atlantic Forest.

## Introduction

Habitat loss and fragmentation can lead to reduction in connectivity of populations with concomitant decrease in gene flow, thus increasing genetic differentiation and reducing genetic variability [1,2]. Genetic diversity is an important component of biodiversity and its reduction can increase the risk of extinction and decrease the evolutionary potential of populations [3,4]. In general, populations that are threatened and their numbers reduced by anthropogenic activities present low levels of genetic diversity resulting from increased genetic drift [5–8]. On the other hand, the low variability found in populations at risk may represent an ancestral state of the population and not a consequence of the recent anthropogenic actions [9]. Differentiating a recent reduction in genetic diversity from an ancestral state of the population can help to plan appropriate conservation measures. This kind of information can be obtained by comparison of historical samples stored in biological collections and extant populations [10–12].

The Harpy Eagle (*Harpia harpyja*, Accipitridae) is the largest eagle in the Neotropical region; it occurs in low densities within its tropical forest habitat [13–15] with more than half its habitat concentrated in Brazil. The Harpy Eagle is a species that depends on the forest, feeds on arboreal prey, nests in emergent canopy trees, returns to the same tree for nesting, and requires large expanses of forest for survival [16,17]. Habitat loss is thus one of the principal threats for the long term survival of the Harpy Eagle and it is the principal reason for the disappearance of the species from most of its range and local extinctions [14]. The tropical forests of the Americas have been drastically reduced in recent decades [18], including in Brazil [19–24]. In the global scenario of habitat loss, the Harpy Eagle is considered Near Threatened with extinction by the International Union for Conservation of Nature [25]. In Brazil, the country with the largest tropical forest area in the world, comprising the Amazon and Atlantic forests, the Harpy Eagle is considered Vulnerable [26]. The cumulative deforestation in the last 45 years has reached approximately 20% of the Brazilian Amazon, and is mainly concentrated in the southern section of the basin [19]. The conservation status of the Harpy Eagle in the Atlantic Forest is precarious since more than 90% of the original forest cover has been lost, with more than half of this loss occurring in the second half of the twentieth century [20]. This has turned the Atlantic Forest into one of the most threatened biodiversity hotspots in the world [27]. Currently, the Harpy Eagle records in the Atlantic Forest are extremely rare [28–31].

Separating the Amazon and the Atlantic Forest is open vegetation composed of the arid thorn bush ‘Chaco’ forest in the southwest, the dry ‘Caatinga’ shrubland in the northeast and the wooded ‘Cerrado’ savanna in the center [32]. However, a series of forest patches and riparian forests create a network of interconnected forests through the open landscape that can serve as habitat and migration corridors for tropical forest species [33–35]. This network provides the possibility of continuity in the distribution or at least can act as corridors for gene flow between the Harpy Eagle of the Amazon and Atlantic forests, and indeed historical and as well as current records of the species in central regions of Brazil exist [36–42]. However, same as with the Amazon and Atlantic forests, the forest patches within the open vegetation corridor separating the two biomes have also been drastically degraded by deforestation [21,22,24].

Among the molecular markers frequently employed for population and conservation genetic studies are the autosomal DNA microsatellites. DNA microsatellites are highly variable, co-dominant markers that can be used to detect changes in the composition of the genetic diversity caused by anthropogenic activities [43]. In this study we employ microsatellite markers to characterize both historical and recent samples of the Harpy Eagle in order to test whether the deforestation of the Brazilian forests negatively impacted the levels and distribution of the genetic diversity of the Harpy Eagle.

## Material and Methods

### Ethics Statement

Permission to collect samples of was granted by System Authorization and Information on Biodiversity SISBIO of the Brazil (Permit Number: 10.3523–1, 10.3523–2, 10.3523–3, 10.3523–4, 15.431–1, 24.960–1 and 24.960–2).

### Sampling

**Fig 1:**
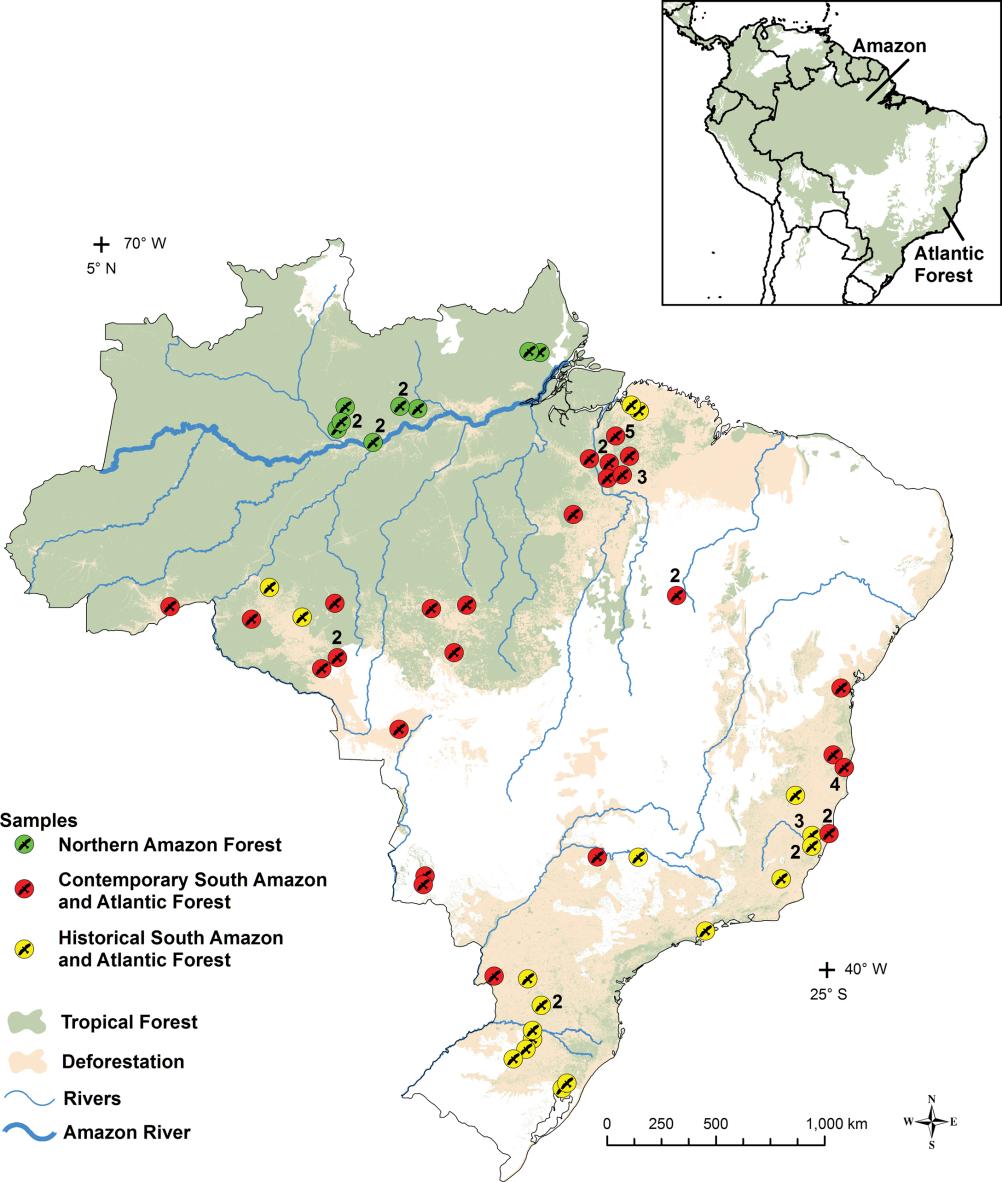
Remnant distribution of tropical forest and deforestation in Brazil with localities of the samples for Northern Amazon, Southern Amazon and Atlantic Forest Historical and Contemporary groups. The circles represent the approximate locations of the analyzed samples and the numbers indicate the number of specimens analyzed in the locality, when more than one. Above is shown the original distribution of tropical forests in Central and South America. Note: Map generated with QGis 2.10.1 Pisa and Inkscape 0.91 software. Data sources: World Wildlife Fund (WWF); Instituto Brasileiro de Geografia e Estatística (IBGE); Fundação SOS Mata Atlântica; Instituto Nacional de Pesquisas Espaciais (INPE); Ministério do Meio Ambiente (MMA); Agência Nacional de Águas (ANA).

Deforestation effects on the genetic diversity of Harpy Eagle was evaluate using samples from historical specimens and samples from areas subject to elevated levels of deforestation in Brazil, corresponding to the southern Amazon (arc of deforestation) and the Atlantic Forest (Fig 1). Amazonian samples from pristine forest in the north of the Amazon River (Fig 1), under less impact of deforestation were considered controls. Analysis was carried out in a total of 72 samples collected from historical and recent/living specimens (contemporary). Only four historical samples were from the Amazon and 18 from the Atlantic Forest (ATF). All samples were derived from the wild, from zoos, from breeders, and from museum or private specimen collections, all originating from Brazilian forests in the period between the years 1904 and 2008 (S1 Table).

Blood, tissue and feather samples were used as source of DNA, with feathers being the most frequently used tissue type. DNA from all samples types was extracted using the Qiagen® DNA Extraction Kit. To degrade keratin of the feathers, 30μl of dithiothreitol (100 mg/ml) was added in the first step of the protocol following the method described by Horváth et al. [44].

### Genotyping

Eight autosomal microsatellite loci transferred to the Harpy Eagle from other species were used. These loci were previously tested, and were found to be polymorphic, in Hardy-Weinberg equilibrium and without linkage disequilibrium [45]. The used loci included NVHfr206 [46], IEAAAG15 [47], HAL09 and HAL10 [48], BBU51 [49], HF-C1D2, HF-C1E8 and HF-C7G4 [50]. The forward *primers* had the M13 primer added at their 5' end, enabling the incorporation of fluorescent dye using the method described by Schuelke [51]. The Polymerase Chain Reaction (PCR) was performed according to the conditions described in Banhos et al [45]. The products of the PCRs were diluted up to 10X. To each 3 μl of the diluted product, 7 μl of formamide and ET-400 Rox (size marker) mixture was added for analysis in the automated DNA sequencer MegaBACE 1000 (GE-Healthcare) following the manufacturer's protocol. The genotypes were visualized and scored in the Fragment Profiler v1.2 software (GE-Healthcare).

The genotyping of museum samples was repeated from two to five times to avoid possible errors due to the small quantities and high levels of degradation of the DNA [52]. Homozygous genotypes from feather samples or uncertain genotypes were also repeated two to three times. Those genotypes that remained uncertain were excluded from analyses. Subsequently, two to ten individuals with homozygous genotypes at any particular locus were sequenced for that locus to confirm the microsatellite sequences of that locus.

### Data Analysis

The program Micro-Checker v2.2 [53] was used to check for null alleles, to identify errors due to large allele dropout and for stutter peaks.

To test whether genetically distinct populations exist, the number of populations (*K*) was estimated using the Bayesian clustering algorithm implemented in Structure 2.3 [54]. Ten independent runs were performed for *K* = 1–5, with 10^6^ MCMC interactions and a 10^5^ burn-in period using the models of correlated allele frequencies and allowing mixture. For each value of *K*, the average log-likelihood was calculated. The analysis was carried out on the complete dataset.

Samples were combined into seven groups for analysis: (i) Northern Amazon (NAM) (N = 11), (ii) Southern Amazon (SAM) (N = 30), (iii) Atlantic Forest (ATF) (N = 31), (iv) Historical Southern Amazon and Atlantic Forest (HSA) (N = 22), (v) Contemporary Southern Amazon and Atlantic Forest (CSA) (N = 39), (vi) Historical Atlantic Forest (HAT) (N = 18) and (vii) Contemporary Atlantic Forest (CAT) (N = 13). Using these groups, we compared several scenarios. Spatial scenarios: NAM, SAM and ATF. Temporal scenarios: HSA and CSA; and HAT and CAT. Spatial-temporal scenarios: NAM, HSA and CSA (Fig 1); and NAM, HAT and CAT.

We used the Arlequin 3.5 software package [55] to assess differences in allele frequencies and gene flow, calculating pairwise pairwise genetic distances (*F*_*ST*_) [56] for the different scenarios; statistical significance of results was tested using 10,000 permutations. The Arlequin 3.5 software was also used to calculate the estimates of genetic diversity: observed heterozygosity (*H*_*O*_) and expected heterozygosity (*H*_*E*_) and the mean number of alleles per locus (*A*).

The Wilcoxon signed-ranks test was employed to test the significance of differences between the levels of genetic variability in the temporal scenarios using the *H*_*E*_ metric as this metric is less sensitive to differences in sample size [57].

To test for recent genetic bottleneck, we tested for heterozygosity excess (*HE*) using the program BOTTLENECK 1.2.02 [58] using the three microsatellites mutation models, the infinite allele model (IAM), stepwise mutation model (SMM) and two-phased model (TPM), assuming that 30% of changes were multistep and 70% followed the stepwise mutation model. Two statistical tests (sign test and Wilcoxon signed-ranks test) were carried out to check whether differences in the expected and observed *HE* values were significant, and thus if there is signal of a recent genetic bottleneck.

## Results and Discussion

The eight microsatellite loci used in this study showed no presence of null alleles, large allele dropout or other deviations when analyzed in Micro-Checker.

Analysis in Structure showed no population structuring; the highest mean probability supported the existence of just one population (LnP (D|*K* = 1) = -1140.30, σ = 19.00; LnP (D|*K* = 2) = -1151.51, σ = 131.35; LnP (D|*K* = 3) = -1262.18, σ = 426.86; LnP (D|*K* = 4) = -1255.54, σ = 418.06; LnP (D|*K* = 5) = -1309.77, σ = 522.97).

In spatial comparisons based on *F*_*ST*_, the pairwise *F*_*ST*_ values were significant between NAM and SAM and in the comparison between NAM and ATF, but was not significant between SAM and ATF (Table 1). These results indicate that the southern Amazon and Atlantic Forest do not harbor distinct populations of the Harpy Eagle and gallery forest connections through the central and southern open vegetation corridor had an important role for maintaining gene flow and that historical connection between the Amazon and Atlantic Forest existed. On the other hand, the significant *F*_*ST*_ values between the northern Amazon and the southern Amazon and the Atlantic Forest suggest the geographic distance or the Amazon River between these regions may be limiting gene flow between populations (e.g., [59,60]), however, this hypothesis has not been tested explicitly in this study.

**Table 1:** Pairwise estimates of genetic differentiation (*F*_*ST*_) and probability (*p*) of observing this differentiation by chance, with sample pairs representing spatial, temporal and temporal-spatial scenarios. Sample group: Northern Amazon (NAM), Southern Amazon (SAM), Atlantic Forest (ATF), Historical Southern Amazon and Atlantic Forest (HSA), Contemporary Southern Amazon and Atlantic Forest (CSA), Historical Atlantic Forest (HAT) and Contemporary Atlantic Forest (CAT).

Sample group pair	*F*_*ST*_	*p*
*Spatial*		
NAM-SAM	0.07599	0.00178*
NAM-ATF	0.07235	0.00000*
ATF-SAM	0.00770	0.11058
*Temporal*		
HSA-CSA	0.02447	0.00693*
HAT-CAT	0.04189	0.00238*
*Temporal-Spatial*		
NAM-HSA	0.06067	0.00089*
NAM-CSA	0.08776	0.00020*
NAM-HAT	0.05661	0.00267*
NAM-CAT	0.11256	0.00000*

*significant at the *p* = 0.05 level.

In temporal comparisons, the differences in allele frequency were significant between HSA and CSA and between HAT and CAT (Table 1). In spatial-temporal comparisons, the differences in *F*_*ST*_ values observed in the comparison between NAM and CSA were greater than those observed between NAM and HSA (Table 1), suggesting that differences in allele frequencies between NAM and southern Amazon and Atlantic Forest is greater currently that it was in the past.

The *H*_*E*_ levels (S2 Table) in the spatial and temporal-spatial comparisons were not significantly different for the eight loci (Tables 2 and 3), although the NAM samples show higher *H*_*E*_ values, despite its smaller sample size. In the temporal comparisons, the *H*_*E*_ level of HSA was approximately 15% higher than in the CSA, with the difference being near significant (Tables 2 and 3). The *H*_*E*_ level of the HAT sample was approximately 19% higher than CAT sample, with the difference being marginally significant (Tables 2 and 3). These results indicate reduction of genetic diversity in the deforested southern Amazonian and Atlantic forests, while the HSA and HAT levels of genetic diversity of Harpy Eagle are similar to the NAM where deforestation pressure is much lower.

**Table 2:** Sample group, number of individuals sampled and diversity estimates: mean observed heterozygosity (*H*_*O*_) and expected heterozygosity (*H*_*E*_), number of alleles per locus (*A*). Sample group: Northern Amazon (NAM), Southern Amazon (SAM), Atlantic Forest (ATF), Historical Southern Amazon and Atlantic Forest (HSA), Contemporary Southern Amazon and Atlantic Forest (CSA), Historical Atlantic Forest (HAT) and Contemporary Atlantic Forest (CAT).

Sample group	Number	*A*	*H*_*O*_	*H*_*E*_
*Spatial*				
NAM	11	3.875	0.6144	0.5690
SAM	30	4.750	0.4483	0.4496
ATF	31	5.000	0.5016	0.4976
*Temporal*				
HSA	22	4.500	0.5566	0.5215
CSA	39	5.250	0.4302	0.4436
HAT	13	4.375	0.5527	0.5379
CAT	18	3.875	0.4342	0.4357

**Table 3:** Pairwise Wilcoxon test (*Z*) and probability (*p*) of observing this differentiation by chance, with sample pairs representing spatial, temporal and temporal-spatial scenarios. Sample group: Northern Amazon (NAM), Southern Amazon (SAM), Atlantic Forest (ATF), Historical Southern Amazon and Atlantic Forest (HSA), Contemporary Southern Amazon and Atlantic Forest (CSA), Historical Atlantic Forest (HAT) and Contemporary Atlantic Forest (CAT).

Sample pair	*Z*	*p*
*Spatial*		
NAM-SAM	1.4000	0.1614
NAM-ATF	1.1200	0.2626
ATF-SAM	1.2600	0.2075
*Temporal*		
HSA-CSA	1.8200	0.0687
HAT-CAT	1.9600	0.0499*
*Temporal-Spatial*		
NAM-HSA	0.8402	0.4008
NAM-CSA	1.5400	0.1234
NAM-HAT	0.8402	0.4008
NAM-CAT	1.5400	0.1234

*significant at the *p* = 0.05 level.

It should be noted that, in general, heterospecific microsatellite loci tend to have lower variability [61] and therefore the markers used in this study may be underestimating the real genetic diversity of the Harpy Eagle. However, this does not seem to be the case, because primers developed specifically for the Harpy Eagle showed a mean *H*_*E*_ = 0.36558 [62] which is lower than the *H*_*E*_ values obtained for primers used in this study.

Despite the population decline the Harpy Eagle is suffering [25], the reduction of the effective population size seems not to have resulted in a temporary excess of heterozygosity detectable in the program Bottleneck (S1 Text).

Because the Harpy Eagle is a top predator with each pair occupying a large home range, its densities are low, and because individuals are very large, the species is rarely found in museum or biological collections. Thus it was not possible to obtain larger and more regular sampling in space and time for the Amazon and Atlantic Forest biomes and this may have influenced the results of some of the analyses. This, combined with a weak signal of incipient differentiation could have resulted in the lack of differentiation in Structure [63]. Otherwise, *F*_*ST*_ values between predefined groups were significant, although apparently small. However, *F*_*ST*_ values less than 0.1 may represent a large genetic divergence, especially when microsatellite markers are employed, and it’s not a statistical issue, deriving from the sampling of individuals from populations [64,65].

Our results were quite different from those encountered in other eagle species. For example, Martínez-Cruz et al. [2] found that population fragmentation led to the present spatial and temporal genetic structure of the Spanish Imperial Eagle (*Aquila adalberti*), with *F*_*ST*_ of 0.12 between current fragmented populations and *F*_*ST*_ of 0.032 between historical and contemporary samples although historical and recent genetic diversity levels were indistinguishable. The Harpy Eagle, as well as other large eagles, has a long lifespan and generation time of 18.5 year [25], thus these life history characteristics may have attenuated the reduction of genetic diversity [66–68], as is thought to have happened with the White Tailed Eagle (*Haliaeetus albicilla*) in Europe [69]. In the case of White Tailed Eagle conservation efforts started in the 1960s with the protection of breeding pairs leading to a wider preservation of genetic diversity in local populations. This kind of conservation effort is likely to be much more efficient in the conservation of the species and preservation of genetic diversity than when conservation efforts focus on a single population [69]. Contrary to the situation experienced by the White Tailed Eagle, threats to the Harpy Eagle intensified in the last 50 years and the actions for the conservation of the species began timidly just over 15 years ago [70]. As seen from the data on the southern Amazon and the Atlantic Forest regions conservation actions are already came too late and are of too small effect to avoid large-scale reduction in genetic diversity in deforested areas.

Genetic diversity levels found in the Brazilian Harpy Eagle are comparable with those found in other eagle species that are not threatened, for example *Haliaeetus albicilla* (*H*_*E*_ = 0.510–0.606) [9,69], *Haliaeetus vocifer* (*H*_*E*_ = 0.413–0.554), *Haliaeetus leucocephalus* (*H*_*E*_ = 0.349–0.464) [9] and *Aquila chrysaetos* (*H*_*E*_ = 0.48) [66]; and some threatened species such as *Aquila adalberti* (*H*_*E*_ = 0.549) and *Aquila heliaca* (*H*_*E*_ = 0.627) [71]. The expected heterozygosity of the Harpy Eagle is much higher than the level of diversity found for *Haliaeetus vociferoides* (*H*_*E*_ = 0.101–0.163) whose reduced genetic diversity is the result of a historical setting of hundreds of thousands of years in which the species went through with a low population [9].

Lerner et al. [72] studied the distribution of the genetic variability of the Harpy Eagle in Central and South America with data from the mtDNA control region. They argued that the high levels of genetic diversity compared to other taxa of the Accipitridae may be explained by the long lifespan of the Harpy Eagle. Lerner et al. [72] collected 66 samples and found greater genetic diversity in South America, emphasizing that the genetic diversity of this region may be underestimated as the region was not well sampled. Moreover, these authors used only two samples from Brazil, which is a region that covers more than 50% of the distribution of the species. The study [72] analyzed examples from tropical forests of Central and other regions of South America which are also being deforested, however, they did not observe reduction in genetic diversity with the marker used. The use of different markers and different experimental design likely explains the differences in the results: no reduction in genetic diversity [72] vs. reduction in genetic diversity (this study). This led to a more optimistic interpretation [72] of what can be done for the Harpy Eagle, in the deforestation scenario of Brazilian tropical forests.

The consequences of the reduction in genetic diversity make the results of this study very preoccupying for the conservation of the Harpy Eagle in southern Amazon and Atlantic Forest. Reduced heterozygosity is a hallmark of populations with reduced fitness and elevated risk of extinction [7]. A correlation of heterozygosity and fitness is positive even if estimated on the basis of microsatellite loci which are assumed to be neutral [73]. The standing diversity is likely to be reduced even further in the future as stochastic genetic processes bring the standing genetic variation to a new equilibrium [3]. Additionally, deforestation continues [19,20] and projections of forest cover loss in the Amazon are alarming [74,75]. The forecast is a loss of habitat of Harpy Eagle from 27.6 to 55.5% by 2057, with majority of the habitat loss concentrated in the arc of deforestation [76]. Beyond the loss of habitat, the Harpy Eagle suffers from effects of illegal hunting and capture for captive breeding, felling of trees with active nests, direct impacts of road construction and associated occupation of areas, hydropower and power transmission network [14,25,70,76–81], all threats that are dramatically reduce their population.

## Conclusions

The use of samples derived from collections allowed us to evaluate the genetic variability of both historical and current samples of the Harpy Eagle in the context of the deforestation scenario of Brazilian tropical forests. The Harpy Eagle genetic diversity that was apparently homogeneous throughout its distribution in the forests of Brazil in the past has become reduced under the current deforestation scenario. However, we believe that conservation measures together with habitat preservation can be positive for the maintenance of the genetic diversity of the Harpy Eagle. Such actions might involve: (i) protection of nesting trees and breeding pairs; (ii) protection of the remaining areas of southern Amazon and Atlantic Forest; and (iii) restoration and protection of forest corridors between the Amazon and Atlantic Forest.

## Supporting Information

S1 Table Information about 72 samples analyzed in this work. (DOCX)

S2 Table Observed heterozygosity (*H*_*O*_) and expected heterozygosity (*H*_*E*_) for locus microsatellite in each sample group. (DOCX)

S1 Text Genetic analysis of population bottleneck in the Harpy Eagle. (DOCX)
