# Supplementary material for: Reduction of Genetic Diversity of the Harpy Eagle in Brazilian Tropical Forests
Source: PLoS One. 2016 Feb 12;11(2):e0148902. doi: 10.1371/journal.pone.0148902 (PMC4752245; doi:10.1371/journal.pone.0148902)
Supplement: S1 Table — (DOCX) [file pone.0148902.s001.docx]

**S1 Table. Information about 72 samples analyzed in this work.**

| Locality - State | Year | Sample Type | Source Nome | Spatial Sample | Temporal Sample |
| --- | --- | --- | --- | --- | --- |
| Alta Floresta - Mato Grosso | 1985 | feather | Zoológico da Universidade Federal de Mato Grosso | SAM | CSA |
| Alto Parnaíba - Maranhão | 2007 | feather | Associação NORDESTA Reflorestamento e Educação | SAM | CSA |
| Alto Parnaíba - Maranhão | 2007 | feather | Associação NORDESTA Reflorestamento e Educação | SAM | CSA |
| Aripuanã - Mato Grosso | 2008 | feather | Centro de Pesquisa para a Conservação das Aves de Rapina Neotropicais - S.O.S Falconiformes | SAM | CSA |
| Bahia | 1997 | feather | CRAX Sociedade de Pesquisa da Fauna Silvestre | ATF | CSA and CAT |
| Bahia | >2000 | feather | Programa de Conservação do Gavião-real | ATF | CSA and CAT |
| Bahia | >2000 | feather | CRAX Sociedade de Pesquisa da Fauna Silvestre | ATF | CSA and CAT |
| Bahia | >1990 | feather | Criadouro Conservacionista Fazenda São José | ATF | CSA and CAT |
| Barra do Bugres - Mato Grosso | 2004 | feather | Programa de Conservação do Gavião-real | SAM | CSA |
| Bonito - Mato Grosso do Sul | 2005 | feather | Parque Nacional da Serra do Bodoquena | ATF | CSA and CAT |
| Campo Novo - Rondônia | 2006 | feather | Programa de Conservação do Gavião-real | SAM | CSA |
| Capim - Pará | 1959 | feather | Museu de Zoologia da Universidade de São - MZUSP 43864 | SAM | HSA |
| Céu Azul - Paraná | ~1982 | feather | Museu de História Natural Capão da Imbuia - MHNCI 2918 | ATF | CSA and CAT |
| Chupinguaia - Rondônia | 2007 | feather | Programa de Conservação do Gavião-real | SAM | CSA |
| Colatina - Espírito Santo | 1958 | feather | Museu Lorenzutti - ML 0361 | ATF | HSA and HAT |
| Colatina - Espírito Santo | 1953 | feather | Museu Nacional - MRFF 424 | ATF | HSA and HAT |
| Colatina - Espírito Santo | 1941 | feather | Museu Nacional - MN 39629 | ATF | HSA and HAT |
| Cupixi - Amapá | 2007 | feather | Reserva Particular do Patrimônio Natural Revecom | NAM |  |
| Faro - Pará | 2004 | muscle | Programa de Conservação do Gavião-real | NAM |  |
| Getúlio Vargas - Rio Grande do Sul | <1950 | feather | Museu Regional Olívio Otto | ATF | HSA and HAT |
| Goianésia - Pará | >1995 | feather | Fundação Zoobotânica de Marabá | SAM | CSA |
| Goianésia - Pará | 2004 | feather | Programa de Conservação do Gavião-real | SAM | CSA |
| Gravataí - Rio Grande do Sul | 1938 | feather | Museu Anchieta de Ciências Naturais | ATF | HSA and HAT |
| Guarantã do Norte - Mato Grosso | 1999 | feather | Zoológico da Universidade Federal de Mato Grosso | SAM | CSA |
| Itacoatiara - Amazonas | 2000 | feather | Instituto Nacional de Pesquisas da Amazônia - INPA 630 | NAM |  |
| Itacoatiara - Amazonas | 1986 | feather | Instituto Nacional de Pesquisas da Amazônia - INPA 588 | NAM |  |
| Itagimirim - Bahia | 2008 | blood | Reserva Particular do Patrimônio Natural Estação Veracel | ATF | CSA and CAT |
| Itagimirim - Bahia | 2008 | blood | Reserva Particular do Patrimônio Natural Estação Veracel | ATF | CSA and CAT |
| Itaperuna - Rio de Janeiro | 1943 | feather | Museu Nacional - MN MF470 | ATF | HSA and HAT |
| Ituverava - São Paulo | 1911 | feather | Museu de Zoologia da Universidade de São Paulo - MZUSP 8261 | ATF | HSA and HAT |
| Jacundá – Pará | 2006 | feather | Parque Zoobotânico Vale | SAM | CSA |
| Laranjeiras do Sul - Paraná | 1964 | feather | Museu de História Natural Capão da Imbuia - MHNCI 2893 | ATF | HSA and HAT |
| Linhares - Espírito Santo | 1970 | feather | Instituto Nacional da Mata Atlântica - MBML 6761 | ATF | CSA and CAT |
| Linhares - Espírito Santo | 1997 | feather | Museu Lorenzutti - ML 0353 | ATF | CSA and CAT |
| Manaus – Amazonas | 2001 | feather | Instituto Nacional de Pesquisas da Amazônia - INPA 828 | NAM |  |
| Nhamundá - Amazonas | 2006 | blood | Instituto Brasileiro do Meio Ambiente e dos Recursos Naturais Renováveis – IBAMA, Amazonas | NAM |  |
| Nhamundá - Amazonas | 2007 | muscle | Instituto Brasileiro do Meio Ambiente e dos Recursos Naturais Renováveis – IBAMA, Amazonas | NAM |  |
| Palestina - São Paulo | ~1970 | feather | Instituto de Biociências, Letras e Ciências Exatas - IBILCE, Campus São José do Rio Preto, Universidade Estadual Paulista Júlio de Mesquita Filho | ATF | CSA and CAT |
| Palmas – Paraná | 1938 | feather | Museu de História Natural Capão da Imbuia - MHNCI 2006 | ATF | HSA and HAT |
| Palmas - Paraná | 1925 | feather | Museu de Zoologia da Universidade de São Paulo - MZUSP 11187 | ATF | HSA and HAT |
| Paragominas – Pará | >1995 | feather | Bioparque Amazônia Crocodilo Safari Zoo | SAM | CSA |
| Paragominas - Pará | >1995 | feather | Bioparque Amazônia Crocodilo Safari Zoo | SAM | CSA |
| Paragominas – Pará | >1995 | feather | Bioparque Amazônia Crocodilo Safari Zoo | SAM | CSA |
| Paragominas - Pará | >1995 | feather | Bioparque Amazônia Crocodilo Safari Zoo | SAM | CSA |
| Paragominas - Pará | >1995 | feather | Bioparque Amazônia Crocodilo Safari Zoo | SAM | CSA |
| Parque Nacional da Serra do Bodoquena - Mato Grosso do Sul | 2007 | feather | Parque Nacional da Serra do Bodoquena | ATF | CSA and CAT |
| Passo Fundo - Rio Grande do Sul | <1950 | feather | Museu de Ciências Naturais da Fundação Zoobotânica do Rio Grande do Sul - MCN 088 | ATF | HSA and HAT |
| Porto Seguro - Bahia | 1997 | feather | Reserva Particular do Patrimônio Natural Estação Veracel | ATF | CSA and CAT |
| Presidente Figueiredo – Amazonas | >1995 | Skin | Programa de Conservação do Gavião-real | NAM |  |
| Presidente Tancredo Neves – Bahia | >1990 | feather | Parque Zoobotânico de Salvador | ATF | CSA and CAT |
| Rio Guamá – Pará | 1904 | feather | Museu Paraense Emílio Goeldi - MPEG 3445 | SAM | HSA |
| Rio Jamary – Rondônia | ~1914 | feather | Museu Nacional - MN 9264 | SAM | HSA |
| Rio Ji-Paraná - Rondônia | ~1914 | feather | Museu Nacional - MN 9265 | SAM | HSA |
| Rio Mucuri - Minas Gerais | <1940 | feather | Museu Nacional - MN 32531 | ATF | HSA and HAT |
| Rondon do Pará – Pará | 1996 | feather | CRAX Sociedade de Pesquisa da Fauna Silvestre | SAM | CSA |
| Rondon do Pará – Pará | 1996 | feather | CRAX Sociedade de Pesquisa da Fauna Silvestre | SAM | CSA |
| Rondon do Pará – Pará | >1995 | feather | CRAX Sociedade de Pesquisa da Fauna Silvestre | SAM | CSA |
| Santa Teresa - Espírito Santo | 1945 | feather | Instituto Nacional da Mata Atlântica - MBML 2097 | ATF | HSA and HAT |
| Santa Teresa - Espírito Santo | 1949 | feather | Instituto Nacional da Mata Atlântica - MBML 6762 | ATF | HSA and HAT |
| Senador Guiomard - Acre | 2006 | feather | Parque Municipal Chico Mendes | SAM | CSA |
| Serra Pelada – Pará | >1995 | feather | Parque das Aves | SAM | CSA |
| Sertão do Puruba - São Paulo | 1967 | feather | Museu de História Natural de Taubaté - MHNT 6720 | ATF | HSA and HAT |
| Sinop - Mato Grosso | 2004 | feather | Parque Ecológico Municipal de Americana Cid Almeida Franco | SAM | CSA |
| Tailândia – Pará | 2004 | feather | Programa de Conservação do Gavião-real | SAM | CSA |
| Tapera, Selbach - Rio Grande do Sul | 1940 | feather | Colégio La Salle Dores | ATF | HSA and HAT |
| Taquara - Rio Grande do Sul | 1923 | feather | Museu de Ciências Naturais da Fundação Zoobotânica do Rio Grande do Sul - MCN 072 | ATF | HSA and HAT |
| Três Arroios - Rio Grande do Sul | 1927 | feather | Museu Frei Miguel | ATF | HSA and HAT |
| Tucuruí – Pará | 2006 | feather | Programa de Conservação do Gavião-real | SAM | CSA |
| Vilhena – Rondônia | >1995 | feather | Parque Dois Irmãos | SAM | CSA |
| Vilhena – Rondônia | >1995 | feather | Parque Dois Irmãos | SAM | CSA |

Sample group: Northern of the Amazon River (NAM), South Amazon (SAM), Atlantic Forest (ATF), Historical South Amazon and Atlantic Forest (HSA), Contemporary South Amazon and Atlantic Forest (CSA), Historical Atlantic Forest (HAT) and Contemporary Atlantic Forest (CAT).

Symbol: ~ approximately,< before than and > after than
