# Supplementary material for: Reduction of Genetic Diversity of the Harpy Eagle in Brazilian Tropical Forests
Source: PLoS One. 2016 Feb 12;11(2):e0148902. doi: 10.1371/journal.pone.0148902 (PMC4752245; doi:10.1371/journal.pone.0148902)
Supplement: S2 Table — (DOCX) [file pone.0148902.s002.docx]

**S2 Table. Observed heterozygosity (***H_O_***) and expected heterozygosity (***H_E_***) for locus microsatellite in each sample group.**

| **Locus** | **Sample group** | | | | | | | | | | | | | |
| --- | --- | --- | --- | --- | --- | --- | --- | --- | --- | --- | --- | --- | --- | --- |
|  | **NAM** | | **SAM** | | **ATF** | | **HSA** | | **CSA** | | **HAT** | | **CAT** | |
|  | ***H_O_*** | ***H_E_*** | ***H_O_*** | ***H_E_*** | ***H_O_*** | ***H_E_*** | ***H_O_*** | ***H_E_*** | ***H_O_*** | ***H_E_*** | ***H_O_*** | ***H_E_*** | ***H_O_*** | ***H_E_*** |
| NVHFR206 | 0.60000 | 0.69474 | 0.73333 | 0.73842 | 0.79310 | 0.79068 | 0.75000 | 0.80513 | 0.76923 | 0.75624 | 0.75000 | 0.80847 | 0.84615 | 0.74154 |
| IEAAAG15 | 1.00000 | 0.86316 | 0.88000 | 0.90122 | 0.67742 | 0.82602 | 0.70000 | 0.81795 | 0.80556 | 0.88459 | 0.72222 | 0.82381 | 0.61538 | 0.84923 |
| HAL09 | 0.54545 | 0.51948 | 0.60000 | 0.53559 | 0.70968 | 0.53094 | 0.77273 | 0.4852 | 0.58974 | 0.55445 | 0.77778 | 0.48889 | 0.61538 | 0.59077 |
| HAL10 | 0.27273 | 0.25541 | 0.03704 | 0.03704 | 0.17241 | 0.22263 | 0.15000 | 0.22949 | 0.08333 | 0.08177 | 0.17647 | 0.2656 | 0.16667 | 0.16304 |
| BBU51 | 0.60000 | 0.46842 | 0.21429 | 0.26039 | 0.19355 | 0.22845 | 0.31818 | 0.33298 | 0.13514 | 0.17882 | 0.27778 | 0.32222 | 0.07692 | 0.07692 |
| HFC1D2 | 0.72727 | 0.73593 | 0.63333 | 0.57175 | 0.76667 | 0.59944 | 0.95238 | 0.65157 | 0.56410 | 0.52681 | 0.94118 | 0.67914 | 0.53846 | 0.44308 |
| HFC1E8 | 0.62500 | 0.60000 | 0.14286 | 0.17013 | 0.20000 | 0.29209 | 0.14286 | 0.29733 | 0.18919 | 0.20030 | 0.17647 | 0.35651 | 0.23077 | 0.21231 |
| HFC7G4 | 0.54545 | 0.41558 | 0.34615 | 0.38235 | 0.50000 | 0.49091 | 0.66667 | 0.55238 | 0.30556 | 0.36581 | 0.60000 | 0.55862 | 0.38462 | 0.40923 |

Northern of the Amazon River (NAM), Southern Amazon (SAM), Atlantic Forest (ATF), Historical Southern Amazon and Atlantic Forest (HSA), Contemporary Southern Amazon and Atlantic Forest (CSA), Historical Atlantic Forest (HAT) and Contemporary Atlantic Forest (CAT).
