# Supplementary material for: Reduction of Genetic Diversity of the Harpy Eagle in Brazilian Tropical Forests
Source: PLoS One. 2016 Feb 12;11(2):e0148902. doi: 10.1371/journal.pone.0148902 (PMC4752245; doi:10.1371/journal.pone.0148902)
Supplement: S1 Text — (DOCX) [file pone.0148902.s003.docx]

**S1 Text. Genetic analysis of population bottlenecking in the Harpy Eagle.**

**Northern of the Amazon River (NAM)**

SIGN TEST

Assumptions: all loci fit I.A.M., mutation-drift equilibrium.

Expected number of loci with heterozygosity excess: 4.36

2 loci with heterozygosity deficiency and 6 loci with heterozygosity excess.

Probability: 0.20888

Assumptions: all loci fit T.P.M., mutation-drift equilibrium.

Expected number of loci with heterozygosity excess: 4.58

3 loci with heterozygosity deficiency and 5 loci with heterozygosity excess.

Probability: 0.53035

Assumptions: all loci fit S.M.M., mutation-drift equilibrium.

Expected number of loci with heterozygosity excess: 4.59

3 loci with heterozygosity deficiency and 5 loci with heterozygosity excess.

Probability: 0.53213

WILCOXON TEST

Assumptions: all loci fit I.A.M., mutation-drift equilibrium.

Probability (one tail for H deficiency): 0.76953

Probability (one tail for H excess): 0.27344

Probability (two tails for H excess and deficiency): 0.54688

Assumptions: all loci fit T.P.M., mutation-drift equilibrium.

Probability (one tail for H deficiency): 0.62891

Probability (one tail for H excess): 0.42188

Probability (two tails for H excess or deficiency): 0.84375

Assumptions: all loci fit S.M.M., mutation-drift equilibrium.

Probability (one tail for H deficiency): 0.52734

Probability (one tail for H excess): 0.52734

Probability (two tails for H excess or deficiency): 1.00000

=================================================================

**Southern Amazon (SAM)**

SIGN TEST

Assumptions: all loci fit I.A.M., mutation-drift equilibrium.

Expected number of loci with heterozygosity excess: 4.20

3 loci with heterozygosity deficiency and 5 loci with heterozygosity excess.

Probability: 0.41790

Assumptions: all loci fit T.P.M., mutation-drift equilibrium.

Expected number of loci with heterozygosity excess: 4.44

4 loci with heterozygosity deficiency and 4 loci with heterozygosity excess.

Probability: 0.51153

Assumptions: all loci fit S.M.M., mutation-drift equilibrium.

Expected number of loci with heterozygosity excess: 4.51

4 loci with heterozygosity deficiency and 4 loci with heterozygosity excess.

Probability: 0.49057

WILCOXON TEST

Assumptions: all loci fit I.A.M., mutation-drift equilibrium.

Probability (one tail for H deficiency): 0.47266

Probability (one tail for H excess): 0.57813

Probability (two tails for H excess and deficiency): 0.94531

Assumptions: all loci fit T.P.M., mutation-drift equilibrium.

Probability (one tail for H deficiency): 0.32031

Probability (one tail for H excess): 0.72656

Probability (two tails for H excess or deficiency): 0.64063

Assumptions: all loci fit S.M.M., mutation-drift equilibrium.

Probability (one tail for H deficiency): 0.15625

Probability (one tail for H excess): 0.87500

Probability (two tails for H excess or deficiency): 0.31250

=================================================================

**Atlantic Forest (ATF)**

SIGN TEST

Assumptions: all loci fit I.A.M., mutation-drift equilibrium.

Expected number of loci with heterozygosity excess: 4.38

3 loci with heterozygosity deficiency and 5 loci with heterozygosity excess.

Probability: 0.46969

Assumptions: all loci fit T.P.M., mutation-drift equilibrium.

Expected number of loci with heterozygosity excess: 4.57

4 loci with heterozygosity deficiency and 4 loci with heterozygosity excess.

Probability: 0.47427

Assumptions: all loci fit S.M.M., mutation-drift equilibrium.

Expected number of loci with heterozygosity excess: 4.61

5 loci with heterozygosity deficiency and 3 loci with heterozygosity excess.

Probability: 0.21164

WILCOXON TEST

Assumptions: all loci fit I.A.M., mutation-drift equilibrium.

Probability (one tail for H deficiency): 0.62891

Probability (one tail for H excess): 0.42188

Probability (two tails for H excess and deficiency): 0.84375

Assumptions: all loci fit T.P.M., mutation-drift equilibrium.

Probability (one tail for H deficiency): 0.27344

Probability (one tail for H excess): 0.76953

Probability (two tails for H excess or deficiency): 0.54688

Assumptions: all loci fit S.M.M., mutation-drift equilibrium.

Probability (one tail for H deficiency): 0.09766

Probability (one tail for H excess): 0.96289

Probability (two tails for H excess or deficiency): 0.19531

=================================================================

**Historical Southern Amazon and Atlantic Forest (HSA)**

SIGN TEST

Assumptions: all loci fit I.A.M., mutation-drift equilibrium.

Expected number of loci with heterozygosity excess: 4.29

3 loci with heterozygosity deficiency and 5 loci with heterozygosity excess.

Probability: 0.44325

Assumptions: all loci fit T.P.M., mutation-drift equilibrium.

Expected number of loci with heterozygosity excess: 4.42

3 loci with heterozygosity deficiency and 5 loci with heterozygosity excess.

Probability: 0.48287

Assumptions: all loci fit S.M.M., mutation-drift equilibrium.

Expected number of loci with heterozygosity excess: 4.52

3 loci with heterozygosity deficiency and 5 loci with heterozygosity excess.

Probability: 0.51150

WILCOXON TEST

Assumptions: all loci fit I.A.M., mutation-drift equilibrium.

Probability (one tail for H deficiency): 0.80859

Probability (one tail for H excess): 0.23047

Probability (two tails for H excess and deficiency): 0.46094

Assumptions: all loci fit T.P.M., mutation-drift equilibrium.

Probability (one tail for H deficiency): 0.42188

Probability (one tail for H excess): 0.62891

Probability (two tails for H excess or deficiency): 0.84375

Assumptions: all loci fit S.M.M., mutation-drift equilibrium.

Probability (one tail for H deficiency): 0.37109

Probability (one tail for H excess): 0.67969

Probability (two tails for H excess or deficiency): 0.74219

=================================================================

**Contemporary Southern Amazon and Atlantic Forest (CSA)**

SIGN TEST

Assumptions: all loci fit I.A.M., mutation-drift equilibrium.

Expected number of loci with heterozygosity excess: 4.39

4 loci with heterozygosity deficiency and 4 loci with heterozygosity excess.

Probability: 0.52618

Assumptions: all loci fit T.P.M., mutation-drift equilibrium.

Expected number of loci with heterozygosity excess: 4.57

4 loci with heterozygosity deficiency and 4 loci with heterozygosity excess.

Probability: 0.47433

Assumptions: all loci fit S.M.M., mutation-drift equilibrium.

Expected number of loci with heterozygosity excess: 4.56

6 loci with heterozygosity deficiency and 2 loci with heterozygosity excess.

Probability: 0.07027

WILCOXON TEST

Assumptions: all loci fit I.A.M., mutation-drift equilibrium.

Probability (one tail for H deficiency): 0.32031

Probability (one tail for H excess): 0.72656

Probability (two tails for H excess and deficiency): 0.64063

Assumptions: all loci fit T.P.M., mutation-drift equilibrium.

Probability (one tail for H deficiency): 0.15625

Probability (one tail for H excess): 0.87500

Probability (two tails for H excess or deficiency): 0.31250

Assumptions: all loci fit S.M.M., mutation-drift equilibrium.

Probability (one tail for H deficiency): 0.03711

Probability (one tail for H excess): 0.97266

Probability (two tails for H excess or deficiency): 0.07422

=================================================================

**Historical Atlantic Forest (HAT)**

SIGN TEST

Assumptions: all loci fit I.A.M., mutation-drift equilibrium.

Expected number of loci with heterozygosity excess: 4.34

3 loci with heterozygosity deficiency and 5 loci with heterozygosity excess.

Probability: 0.45966

Assumptions: all loci fit T.P.M., mutation-drift equilibrium.

Expected number of loci with heterozygosity excess: 4.47

3 loci with heterozygosity deficiency and 5 loci with heterozygosity excess.

Probability: 0.49705

Assumptions: all loci fit S.M.M., mutation-drift equilibrium.

Expected number of loci with heterozygosity excess: 4.52

3 loci with heterozygosity deficiency and 5 loci with heterozygosity excess.

Probability: 0.51326

WILCOXON TEST

Assumptions: all loci fit I.A.M., mutation-drift equilibrium.

Probability (one tail for H deficiency): 0.84375

Probability (one tail for H excess): 0.19141

Probability (two tails for H excess and deficiency): 0.38281

Assumptions: all loci fit T.P.M., mutation-drift equilibrium.

Probability (one tail for H deficiency): 0.47266

Probability (one tail for H excess): 0.57813

Probability (two tails for H excess or deficiency): 0.94531

Assumptions: all loci fit S.M.M., mutation-drift equilibrium.

Probability (one tail for H deficiency): 0.37109

Probability (one tail for H excess): 0.67969

Probability (two tails for H excess or deficiency): 0.74219

=================================================================

**Contemporary Atlantic Forest (CAT)**

SIGN TEST

Assumptions: all loci fit I.A.M., mutation-drift equilibrium.

Expected number of loci with heterozygosity excess: 4.33

4 loci with heterozygosity deficiency and 4 loci with heterozygosity excess.

Probability: 0.54271

Assumptions: all loci fit T.P.M., mutation-drift equilibrium.

Expected number of loci with heterozygosity excess: 4.31

6 loci with heterozygosity deficiency and 2 loci with heterozygosity excess.

Probability: 0.09757

Assumptions: all loci fit S.M.M., mutation-drift equilibrium.

Expected number of loci with heterozygosity excess: 4.54

6 loci with heterozygosity deficiency and 2 loci with heterozygosity excess.

Probability: 0.07173

WILCOXON TEST

Assumptions: all loci fit I.A.M., mutation-drift equilibrium.

Probability (one tail for H deficiency): 0.42188

Probability (one tail for H excess): 0.62891

Probability (two tails for H excess and deficiency): 0.84375

Assumptions: all loci fit T.P.M., mutation-drift equilibrium.

Probability (one tail for H deficiency): 0.12500

Probability (one tail for H excess): 0.90234

Probability (two tails for H excess or deficiency): 0.25000

Assumptions: all loci fit S.M.M., mutation-drift equilibrium.

Probability (one tail for H deficiency): 0.01953

Probability (one tail for H excess): 0.98633

Probability (two tails for H excess or deficiency): 0.03906
